# MiR–20a-5p promotes radio-resistance by targeting Rab27B in nasopharyngeal cancer cells

**DOI:** 10.1186/s12935-017-0389-7

**Published:** 2017-03-01

**Authors:** Dabing Huang, Geng Bian, Yueyin Pan, Xinghua Han, Yubei Sun, Yong Wang, Guodong Shen, Min Cheng, Xiang Fang, Shilian Hu

**Affiliations:** 1 Shandong University School of Medicine, Jinan, 250012 China; 2 Department of Geriatrics, Anhui Provincial Hospital, Anhui Medical University, Hefei, 230031 Anhui China; 3 Department of Oncology, Anhui Provincial Hospital, Anhui Medical University, Hefei, 230001 China; 4 Anhui Provincial Key Laboratory of Tumor Immunotherapy and Nutrition Therapy, Hefei, 230001 China

**Keywords:** Rab27B, miR-20a-5p, Radio-resistance, Nasopharyngeal cancer

## Abstract

**Background:**

MicroRNAs (miRNAs) was reported to be involved in cancer radio-resistance, which remains a major obstacle for effective cancer therapy.

**Methods:**

The differently expressed miRNAs were detected by RNA-seq experiment in nasopharyngeal cancer (NPC) cells. MiR-20a-5p was selected as our target, which was subject to finding its target gene Rab27B via bioinformatics analysis. The qRT-PCR, western blot and the luciferase reporter assays were performed to confirm Rab27B as the target of miR-20a-5p. In addition, the roles of miR-20a-5p in NPC radio-resistance were detected by transfection of either miR-20a-5p-mimic or miR-20a-5p-antagomiR. The involvement of Rab27B with NPC radio-resistance was also detected by the experiments with siRNA-mediated repression of Rab27B or over-expression of GFP-Rab27B. Wound healing and invasion assays were performed to detect the roles of both miR-20a-5p and Rab27B.

**Results:**

MiR-20a-5p promotes NPC radio-resistance. We identified that its target gene Rab27B negatively correlates with miR-20a-5p-mediated NPC radio-resistance by systematic studies of a radio-sensitive (CNE-2) and resistant (CNE-1) NPC cell lines. Repression of Rab27B by siRNA suppresses cell apoptosis and passivates CNE-2 cells, whereas over-expression of Rab27B triggered cell apoptosis and sensitizes CNE-1 cells.

**Conclusions:**

MiR-20a-5p and its target gene Rab27B might be involved in the NPC radio-resistance. Thus the key players and regulators involved in this pathway might be the potential targets for developing effective therapeutic strategies against NPC.

**Electronic supplementary material:**

The online version of this article (doi:10.1186/s12935-017-0389-7) contains supplementary material, which is available to authorized users.

## Background

The malignant tumor nasopharyngeal carcinoma (NPC) occurs in the lining of nasopharynx with a multifactorial etiology [1]. Beyond the chemotherapy [2], radiation therapy is the other major methods against cancer due to its excellent local control and increased overall survival rates [3–5]. However, owing to the high sensitivity, radiation therapy often fails in various cancers, such as NPC. The main reason is that radiation treatment can intrinsically induce radio-resistant tumor cells, which show enhanced DNA repair ability [6]. To overcome the problem of radio-resistance, it is urgently needed to elucidate the mechanisms of radio-resistance and develop new radiosensitizers.

MicroRNAs (miRNAs) are non–coding regulatory RNAs, post-transcriptionally regulate gene expression through targeting to a panel of target genes. As the critical roles reported [7], their dysregulation is associated with human diseases, including cancer biology [8, 9]. Notably, the emerging studies have shown that miRNAs are associated with the development of radio-resistance in different type of cancers [10, 11], such as prostate cancer [12], esophageal cancer [13]. As one of the well-studied miRNAs, miR-20a has been shown to function as an oncomiR in many cancers, including lung cancer [14], hepatocellular carcinoma [15], and gastric cancer [16]. Notably, miR-20a was also found to be involved in cancer irradiation treatment [17]. For example, miR-20a was shown to induce cell radio-resistance by activating the PTEN/PI3 K/Akt signaling pathway in hepatocellular carcinoma [18].

In the present study, we performed an RNA-seq assay to detect differentially expressed genes in radio-sensitive (CNE-2) versus radio-resistant (CNE-1) NPC cell lines. We showed that miR-20a-5p promoted NPC radio-resistance via repression of Rab27B, a newly identified target of miR-20a-5p. We further performed a systematic analysis of Rab27B and miR-20a-5p for their roles in the NPC radio-resistance. The regulatory effect of miR-20a-5p on NPC cell survival and apoptosis was also detected upon irradiation.

## Methods

### Cell lines

Human nasopharyngeal cancer cell lines, CNE-1 and CNE-2 were supplied by the department of radiation oncology of Sun Yat-sen University, Guangzhou, China [19]. Cells were cultured in Dulbecco’s modified Eagle’s medium (DMEM) (Gibco, USA) supplemented with 10% fetal bovine serum (Gibco, USA) in a humid atmosphere containing 5% CO_2_ at 37 °C.

### RNA-Seq analysis

RNA-seq analysis was performed by BGI-Tech (Shenzhen, China). RNA was purified and fragmented to construct the RNA-seq library for sequencing. The sense and anti-sense cDNA molecules were synthesized. After agarose gel electrophoresis, suitable fragments were used as templates for PCR amplification. Real-Time PCR System was used in quantification and qualification of the sample library. Finally, the library was subjected to sequencing using Illumina HiSeq 2000 (Illumina, USA). The single-end library was prepared following the protocol of the IlluminaTruSeq RNA Sample Preparation Kit (Illumina) [20].

### Cell reagents

The *Homo sapien* miR–20a-5p mimics, miR–20a-5p antagomiRs and miR–20a-5p scrambled nega-tive control (NC) were obtained from Guangzhou Ribobio, China. All the transfection experiments were performed using the Lipofectamine 2000 transfection reagent (Invitrogen Life Technologies), which was described previously [21]. Western blot and qRT–PCR assays were performed to confirm the effect of Rab27Bon the expression of miR–20a-5p. The sequences used in this study are as follows:

si-Rab27B

5′- CAGUAGGAAUAGACUUUCG dTdT-3′

3′-dTdT GUCAUCCUUAUCUGAAAGC-5′;

hsa-miR-20a-5p

antagomiR: 5′-CUACCUGCACUAUAAGCACUUUA-3′

mimic:

sense 5′-UAAAGUGCUUAUAGUGCAGGUAG-3′

antisense 5′-CUACCUGCACUAUAAGCACUUUA-3′

### Irradiation and clonogenic assay

Cells treated with miRNAs were seeded on 6-well plates in triplicate and exposed to radiation at the doses indicated using a 6-MV x-ray generated by a linear accelerator (Varian trilogy at a dose rate of 200 cGy/min). After incubation at 37 °C for 14 days, cells were fixed in 100% methanol and stained with 0.1% crystal violet. Colonies containing >50 cells were counted under a light microscope. The surviving fraction was calculated as described previously [13, 18]. At least three independent experiments were performed to calculate the means and standard deviations.

### RNA analysis

Total RNA was extracted using Trizol (Vazyme). For the mRNA analysis, the cDNA primed by oligo-dT was made with RT reagent kit (Tiangen, China), and the mRNA level of Rab27B was quantified by a duplex-qRT-PCR analysis where the TaqMan probes with a different fluorescence for β-actin (Shing Gene, China) were used in the FTC-3000P PCR instrument (Funglyn, Canada). The miRNA expression level was normalized using U6 small nuclear RNA (HmiRQP9001) as an internal control, as previ-ously described [22]. Using the 2^−ΔΔCt^ method, the β-actin level was normalized before comparing the relative level of the target genes. The sequences of primers and probes used for the qRT-PCR analysis are as follows:

hRab27BF, 5′-GGGACACTGCGGGACAAG-3′;

hRab27BR, 5′-CAGTTGGCTCATCCAGTTTCTG-3′;

hRab27B probe, 5′-ROX-CGGTTCCGGAGTCTCACCACTGC-3′;

hACTB F: 5′-GCCCATCTACGAGGGGTATG-3′

hACTB R: 5′-GAGGTAGTCAGTCAGGTCCCG-3′

hACTB probe: 5′-CY5-CCCCCATGCCATCCTGCGTC-3′

### Western blotting assays

Total proteins were extracted from cultured cells with cell lysis buffer (60 mM Tris–HCl, pH 6.8, 2% SDS, 20% glycerol, 0.25% bromophenol blue, and 1.25% 2-mercaptoethanol) and heated at 95 °C for 10 min. The heated proteins were separated by 10% SDS-PAGE gel and transferred to polyvinylidene difluoride (PVDF) membranes. After blocking with 5% non-fat milk in TBST for 2 h, the membranes were incubated overnight at 4 °C with diluted Anti-Rab27B primary antibody (13412-1-AP; SanYing, China). Followed by washing with TBST buffer three times, the membranes were incubated with secondary antibody (SA00001-2; SanYing, China) at 37 °C while shaking on a rotary for 2 h. The relative density (level) of proteins over the GAPDH (10494-1-AP; SanYing, China) band was quantified with the Gel-Pro Analyzer (Media Cybernetics).

### Cell apoptosis analysis

Apoptosis was analyzed using Annexin V/PI double staining. After transfection for 48 h, the cells in the logarithmic growth phase were harvested and rinsed twice with ice-bathed PBS, then FITC-labeled enhanced annexin V (3 μl) and propidium iodide (3 μl) were added to the cell suspension at the final volume of 150 μl. After incubation for 30 min, flow cytometry was performed on a FACS Calibur instrument. The number of apoptotic and necrotic cells were calculated by flow cytometry (Becton–Dickinson, USA) and analyzed by Flowjo Software. The ratio of early apoptosis was used for the test results. The experiments were performed three times independently, and a representative is shown.

### Luciferase reporter assay

Cells were seeded in 24-well plate at a concentration of 2 × 10^5^ cells/per well and co-transfected 24 h later with pGL3-luc-Rab27B UTR WTand miR-20a-5pmimic/antagomir or NC. After transfection for 48 h, cells were collected, and the relative luciferase activity was performed using Dula-Luciferase Reporter Assay Kit (Promega). The relative firefly luciferase activities of the UTR construct was analyzed as previously reported [23].

### Wound-healing assays

For cell motility assays, cells stably expressing mimics, antagomiRs or NC were seeded in 24-well plates and cultured to near confluence. After culture for 6 h in DMEM without FBS, a linear wound was carefully made using a sterile 10 µl pipette tip across the confluent cell monolayer, and the cell debris was removed by washing with phosphate-buffered saline. The cells were incubated in DMEM plus 10% FBS, and the wounded monolayers were then photographed at 0, 8, 24 and 48 h after wounding.

### Invasion assays

According to the manufacture’s description, cell invasion assays were performed in a 24-well Transwell Chambers with 8 mm pore size chamber inserts (Corning, USA). In the assay, 1 × 10^4^ cells were seeded into the upper chamber with 200 µl of DMEM without FBS. In the lower chamber, 600 µl of DMEM supplemented with 10% FBS was added. After incubation for 40 h at 37 °C and 5% CO_2_, the non-invading cells were removed from the plate with cotton stick.The cells that moved to the bottom surface were stained with 0.1% crystal violet for 30 min at 37 °C. The cells were then imaged and counted in at least 5 random fields using a CKX41 inverted microscope (Olympus, Tokyo, Japan). The assays were conducted three independent times.

### Immunofluorescent staining for γ-H2AX

Twenty-four hours following transfection with miR-20a-5p mimic or miRNA mimic negative control, 1 × 10^5^ cells were seeded in chamber slides and incubated overnight. The cells were subsequently exposed to 4 Gy irradiation (IR). Twenty-four hours following IR, the cells were fixed in 4% paraformaldehyde, permeabilized in 0.1% Triton X-100 (Sigma), blocked in 2% bovine serum albumin and incubated with a primary antibody against γ-H2AX (SanYing, China) overnight at 4 °C. The primary antibody was subsequently washed off, and a secondary antibody conjugated to fluorescein isothiocyanate was applied to the slides. Cells were washed with phosphate-buffered saline and counterstained with DAPI. The γ-H2AX foci were observed under a fluorescence microscope (Olympus). For each group, the γ-H2AX foci were counted in ≥50 cells.

### Statistical analyses

The data are presented as the mean, and the error bars indicate the SD. All statistical analyses were performed with Excel (Microsoft, Redmond, WA, USA). Two-tailed Student’s *t* test, a one-way analysis of variance or Mann–Whitney U test was used to calculate statistical significance. A *P*-value of <0.05 was considered significant.

## Results

### Rab27B negatively regulates the NPC radio-resistance

**Fig. 1:**
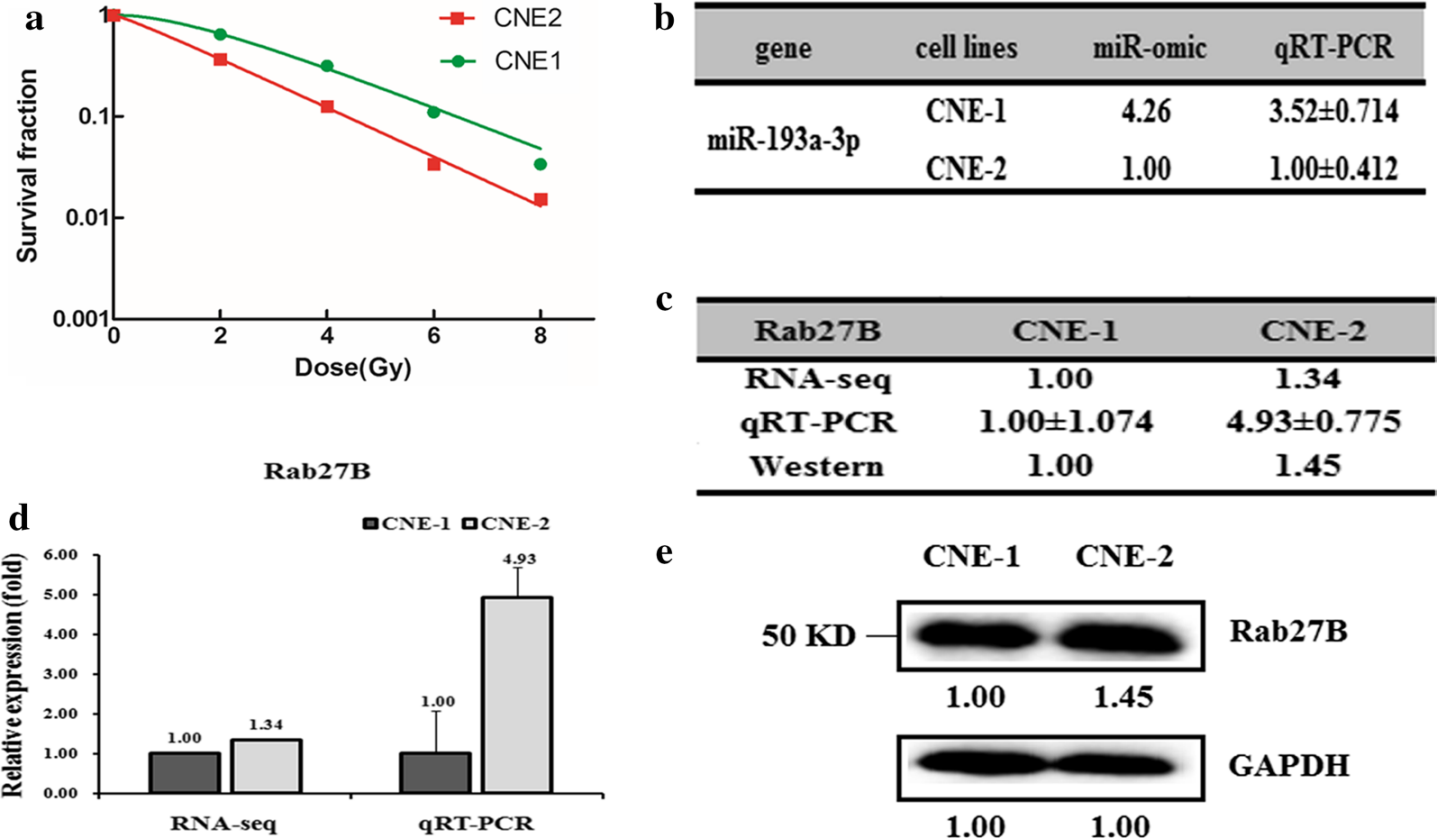
Different expression patterns of miR-20a-5p/Rab27B in nasopharyngealcells CNE-1 and CNE-2. **a** The survival fraction of CNE-1 and CNE-2 NPC cells treated as described. The surviving fraction was calculated using the multitarget single-hit model: Y = 1−(1 − exp(−k*x))^N. The miR-20a-5p expression levels in CNE-1 and CNE-2 cells were analyzed by miR-seq and qRT-PCR analyses in* table* (**b**). The expression level of Rab27B is higher in CNE-2 cells than in CNE-1 cells, as summarized in* table* (**c**). qRT-PCRandWestern blot analyses are shown in *plots* **d** and **e**, respectively

We select two NPC cell lines, CNE-1 and CNE-2 as our targets, which were reported as relatively radio-resistant and radio-sensitive cell lines of nasopharyngeal cancer (NPC), respectively [19, 24–26]. We first detected the radio-sensitivity of these two cell lines and found that CNE-1 cells are more radio-resistant than CNE-2 cells (Fig. 1a). To detect the differentially expressed miRNAs in NPC cells, we performed an RNA-seq assay of CNE-1 and CNE-2 cells (Additional file 1: Table S1). The level of miR-20a-5p was over fourfold higher in CNE-1 cells than that in the CNE-2 cells. We thus further tested the expression of miR-20a-5p by qRT-PCR, which gave a 3.52-fold higher expression in CNE-1 cells (Fig. 1b). Afterwards, we predicted the target genes of miR-20a-5p based on the literature and websites. The predicted target mRNAs were further subject to comparing the expression pattern between CNE-1 and CNE-2 cells by RNA-seq analysis. Dozens of common genes have been found which show a drastically different expression pattern in the two cell lines. Among them, the Rab27B gene is one of the significantly differentially expressed genes that negatively correlate with the expression of miR-20a-5p. Consequently, the expression level of Rab27B was higher in CNE-2 than CNE-1 at both mRNA (RNA-seq based miR-omic: 1.34:1, and qRT-PCR analysis: 4.93:1) and protein level (western blot: 1.45:1) (Fig. 1c–e). The lower expression of Rab27B in radio-resistant CNE-1 cells suggests that Rab27B is a negative regulator of NPC radio-resistance.

### The Rab27B gene is a target of miR-20a-5p in NPC cells

**Fig. 2:**
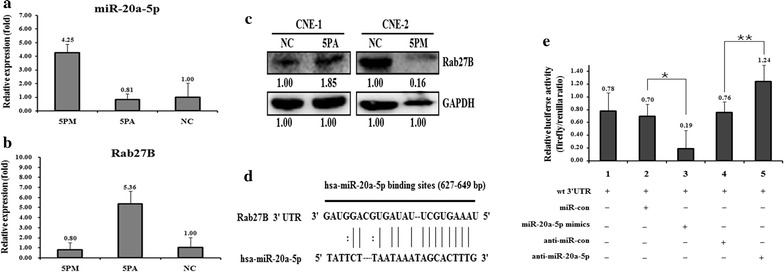
Rab27B is a target of miR-20a-5p in NPC cells. Level of miR-20a-5p,Rab27B mRNA and protein levels in the miR-20a-5p mimic (5PM)-transfected CNE-2 and the miR-20a-5p antagomiR (5PA)-transfected CNE-1 cells versus the negative control (NC) cells, as determined by qRT-PCR (**a**, **b**) and western blot analyses (**c**). The perfectly matched region of Rab27B gene 3'-UTR with miR-20a-5p (**d**). The relative luciferase activity (fold) of the reporter with wild-type (WT) Rab27B-UTR were determined in the miR-20a-5p mimic or anti or Controls transfected NPC cells. The reporter without Rab27B-UTR (Vec) was used as a reference. The Renilla luciferase activity of a co-transfected control plasmid was used to control the transfection efficacy (**e**). The representative results from three independent experiments shown. *P value < 0.05; **P value < 0.01

We found that Rab27B negatively correlates with the level of miR-20a-5p, which indicated that miR-20a-5p might regulate the expression of Rab27B. To check whether Rab27B is one of the authentic targets of miR-20a-5p, we determined the Rab27B level in the miR-20a-5p mimic transfected CNE-2 and the antagomiR transfected CNE-1 cells versus the NC (scramble sequence control) transfected. The transfection of miR-20a-5p mimic in CNE-2 cells increased its expression to over fourfold (Fig. 2a), whereas the transfection of miR-20a-5p antagomiR in CNE-1 cells significantly decreased its level to 81% (Fig. 2a). Following the changes of the miR-20a-5p level, a miR-20a-5p mimic transfection brought down the Rab27B mRNA to 80% (Fig. 2b) and protein to nearly 16% (Fig. 2c) compared to that in the NC transfected CNE-2 cells. As expected, the transfection of miR-20a-5p antagomiR increased the mRNA level of Rab27B by 5.36 fold (Fig. 2b) and the protein level by 1.85 fold in CNE-1cells (Fig. 2c).

Sequence analysis revealed that 3′-UTR region of Rab27B contains one potential binding motif (from 627 to 649 bp) for miR-20a-5p (Fig. 2d). To further conclude whether Rab27B is a direct target of miR-20a-5p, we put the wild-type Rab27B gene at the downstream of the Renilla luciferase gene of pGL3-control vector (Promega) to create pGL3-Rab27B UTR WT (Fig. 2d). These constructs were transfected into CNE-2 and CNE-1 cells, respectively, to compare the luciferase activity. The pGL3-Rab27B-UTR WT gave the relative luciferase activity of 0.78 (Fig. 2e). The transfection of miR-20a-5p-mimic into CNE-2 cells significantly brought down the luciferase activity of pGL3-Rab27B-UTR WT construct, whereas the control cells showed almost the same activity upon the transfection of miR-20a-5p-mimic (Fig. 2e). Meanwhile, the transfection of miR-20a-5p-antagomiR into CNE-1 cells drastically raised the luciferase activity of pGL3-Rab27B-UTR WT construct (Fig. 2e). Getting together, our results strongly indicate that Rab27B is indeed a target of miR-20a-5p.

### The Rab27B expression negatively correlates with the miR-20a-5p’s promoting effect on the NPC radio-resistance

**Fig. 3:**
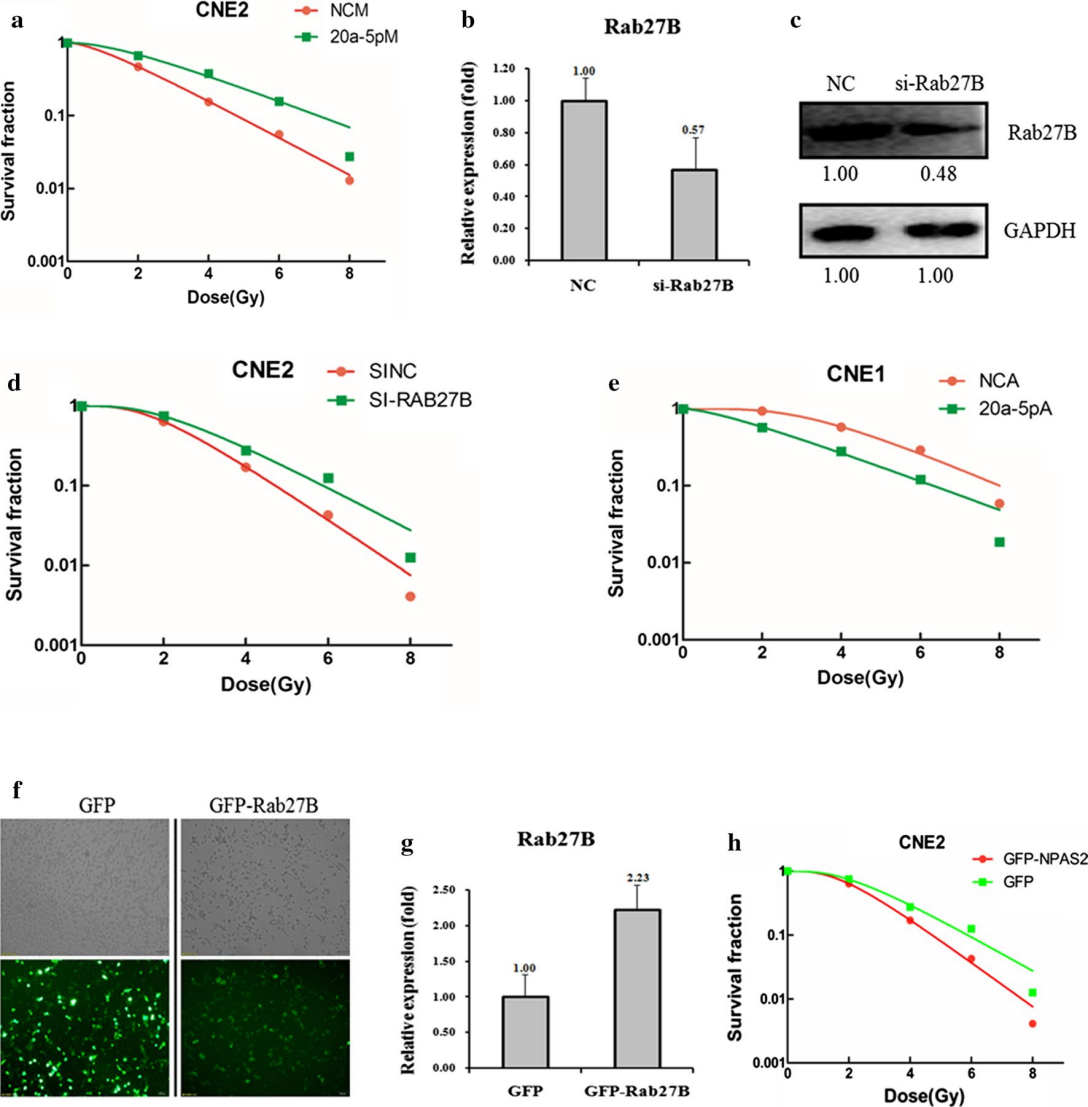
Effects of a forced reversal of the miR-20a-5p or Rab27B levels on the radio-resistance of CNE-1 and CNE-2 cells. MiR-20a-5p mimic (5PM)-transfected CNE-2 increases NC cells resistance to radiation treatment versus the negative control (NC) cells (**a**). Rab27B protein level (*western blot* analysis) and mRNA determined by qRT-PCR in the si-Rab27B-transfected versus the NC-transfected CNE-2 cells treated with a 4-Gy dose of radiation (**b**, **c**). Si-Rab27B-transfected CNE-2 increases NC cells resistance to radiation treatment versus the negative control (NC) cells (**d**). MiR-20a-5p antagomiR (5PA)-transfected CNE-1 decreases NC cells resistance to radiation treatment versus the negative control (NC) cells (**e**). Expression of Rab27B in overexpression construct transfected CNE-1 cells. Representative areas of CNE-1 cells transfected with GFP-Rab27B ectopic expression construct were shown and GFP was used as a negative control (**f**). Rab27B mRNA level (qRT-PCR analysis) in the GFP-Rab27B-transfected versus the GFP-transfected CNE-1 cells treated with a 4-Gy dose of radiation (**g**). GFP- Rab27B-transfected CNE-1 decreases NC cells resistance to radiation treatment versus the negative control (GFP) cells (**h**)

To explore the role of Rab27B in the NPC radio-resistance, we first transfected miR-20a-5p-mimic into CNE-2 cells and tested the level of miR-20a-5p. The transfection of miR-20a-5p-mimic indeed increased the level of miR-20a-5p in the CNE-2 cells. Accompanied by the increase of miR-20a-5p, the cell survival rate was increased against the radiation treatment in the CNE-2 cells (Fig. 3a). In addition, the fluorescent immunostaining against γ-H2AX also showed increased cell viability upon the addition of miR-20a-5p-mimic into the CNE-2 cells (Additional file 2: Figure S1). Consequently, the transfection of miR-20a-5p-mimic into the CNE-2 cells desensitizes NPC cells to irradiation (Additional file 1: Table S1). Then we transfected the si-Rab27B into CNE-2 cells and tested the effect against radiation. The transfection of si-Rab27B into CNE-2 cells indeed decreased the level of Rab27B in both mRNA (0.57:1) and protein level (0.48:1), compared to the control cells (Fig. 3b, c). Consequently, the radio-resistance of CNE-2 cells was also increased with the transfection of si-Rab27B (Fig. 3d). In addition, we transfected miR-20a-5p antagomiR into CNE-1 cells to decrease the level of miR-20a-5p, which results in a lower cell survival rate against irradiation treatment in CNE-1 cells (Fig. 3e). A similar effect was also found in the fluorescent immunostaining assays against γ-H2AX (Additional file 2: Figure S1). Moreover, we over-expressed Rab27B by 2.23 fold (Fig. 3f, g), which also results in a lower cell survival rate against irradiation treatment in CNE-1 cells (Fig. 3h). The results correlate well with the negative regulation of Rab27B in the NPC radio-resistance. Taken together, the Rab27B gene does contribute a great deal to the miR-20a-5p’s promoting effect on the NPC radio-resistance.

### MiR-20a-5p promotes cell migration and invasion of NPC cells

**Fig. 4:**
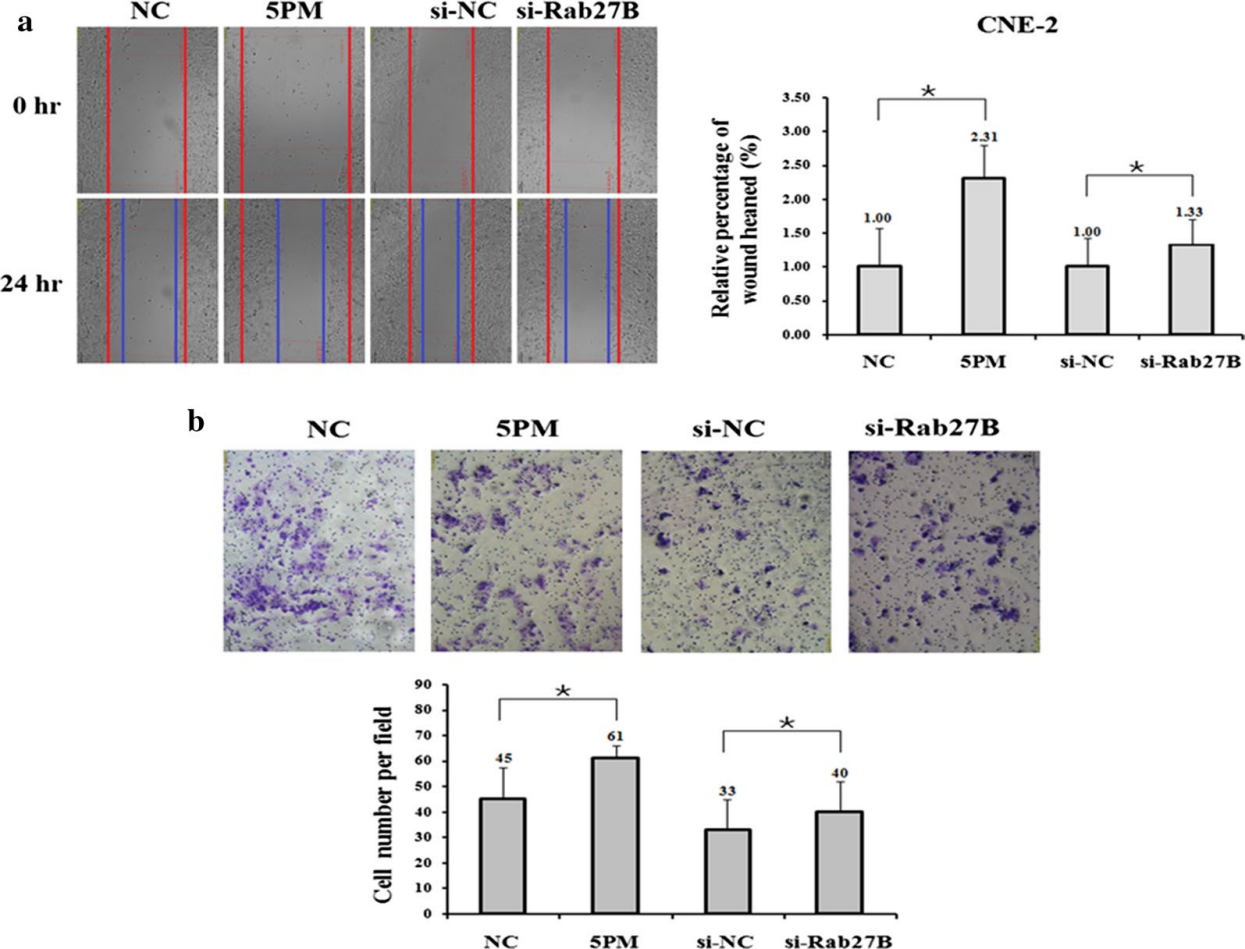
Wound-healing assays was performed with transient expression of the miR-20a-5p mimic (5PM) mimic, si-Rab27B and corresponding negative control (NC) (**a**). Invasion assays was performed with transient expression of the miR-20a-5p mimic (5PM) mimic, si-Rab27B and corresponding negative control (NC) (**b**). The data are representative of three independent experiments. *P < 0.05

**Fig. 5:**
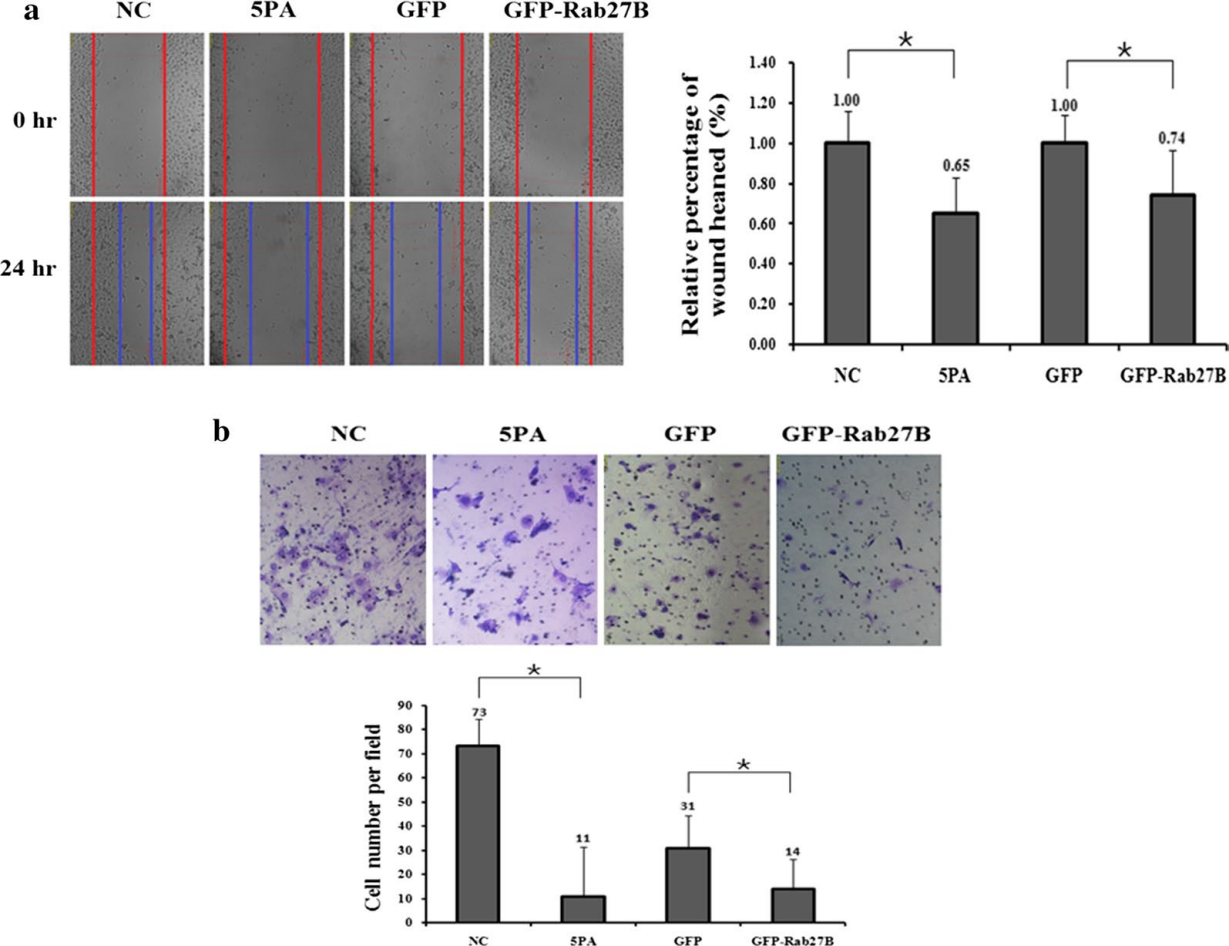
Wound-healing assays was performed with transient expression of the miR-20a-5p antagomiR (5PA), GFP-Rab27B and corresponding negative control (NC) (**a**). Invasion assays was performed with transient expression of the miR-20a-5p antagomiR (5PA) and corresponding negative control (NC) (**b**). The data are representative of three independent experiments. *P < 0.05

To explore whether miR-20a-5p is involved in the metastasis of NPC cells, we compared the migration and invasion capability of CNE-1 and CNE-2 cells using the wound-healing and invasion assays, respectively. We first transfected miR-20a-5p mimics or si-Rab27B into CNE-2 cells and detected the migration and invasion. Compared to the control cells, transfection of miR-20a-5p mimic or si-Rab27B significantly increased the ability of cell migration to about 2.31- and 1.33-fold, similar to that for the invasion assays (Fig. 4a, b). Then we transfected miR-20a-5p antagomiRs or over-expressed GFP-Rab27B into CNE-1 cells and also performed the same experiments. The results showed that both the migration and invasion of CNE-1 cells were decreased to some extent upon the transfection (Fig. 5a, b). All these results suggest that miR-20a-5p enhances the migration and invasion of NPC cells, which might be conducted by the regulation of the Rab27B gene.

**Fig. 6:**
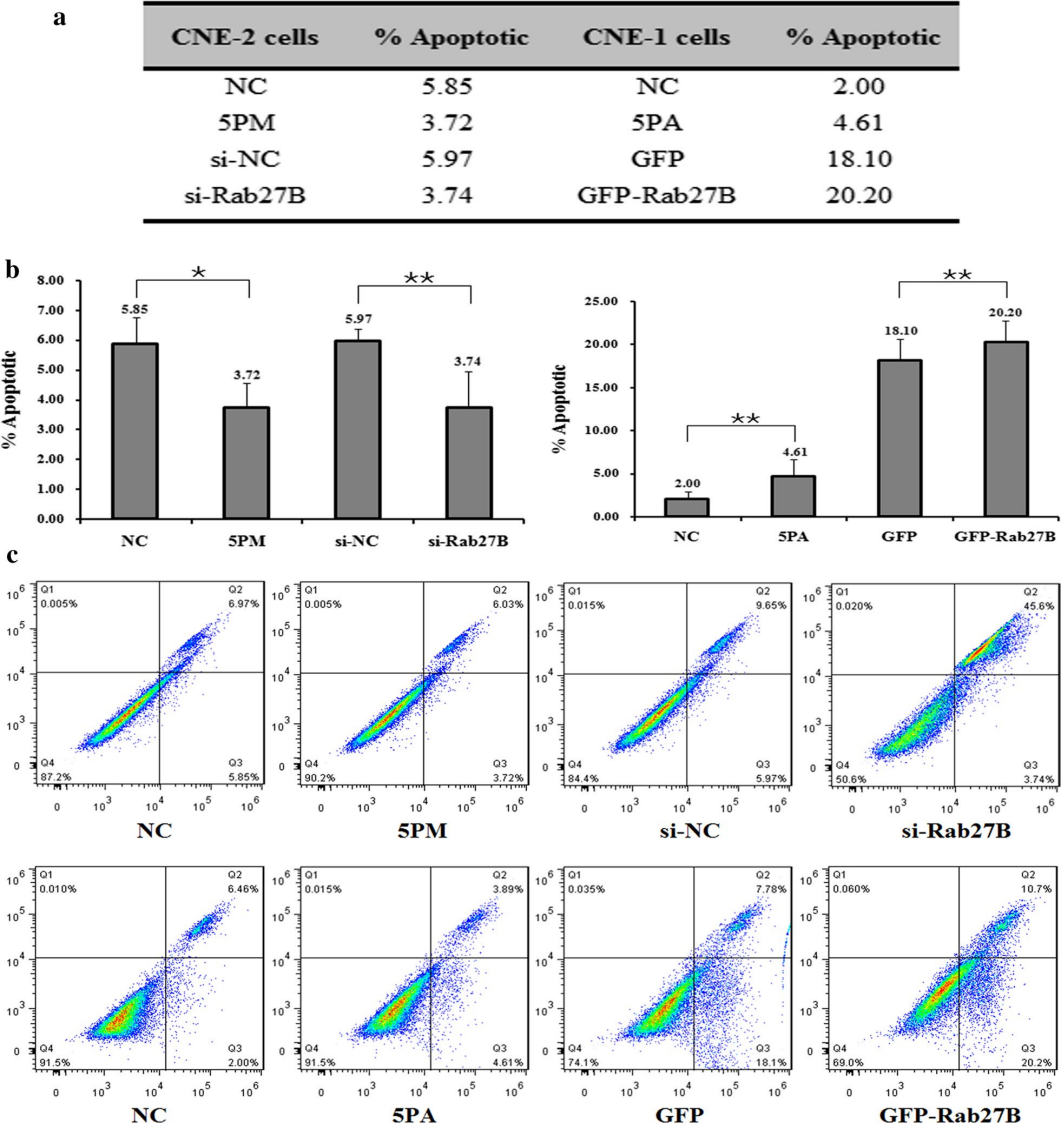
Effects of the forced reversal with transient expression of the miR-20a-5p mimic (5PM), si-Rab27B and corresponding negative control (NC) levels on the apoptosis of CNE-2 cells, and the effects of the forced reversal with transient expression of the miR-20a-5p antagomiR (5PA), GFP-Rab27B and corresponding negative control (NC) levels on the apoptosis of CNE-1 cells, with a graph of the analyzed data (**a**, **b**) and plots of the original FACS data (**c**) *P < 0.05, **P < 0.01

In line with its negative effect of Rab27B on cell migration and invasion, a siRNA-mediated Rab27B repression reduced the cell apoptosis rate from 5.97 to 3.74%, indicating an elevated cell survival rate upon the addition of si-Rab27B into CNE-2 cells (Fig. 6). In line with the apoptosis rate, the expression of the cell apoptosis markers, PARP1 and GAPDH were significantly decreased upon the transfection of si-Rab27B into CNE-2 cells (Additional file 3: Figure S2). A similar effect was also found in the miR-20a-5p-mimic transfected CNE-2 cells (Fig. 6a–c). On the other hand, the transfection of miR-20a-5p-antagomiR slightly increased the cell apoptosis rate in CNE-1 cells, which also suggests a promoting effect of Rab27B on NPC radio-resistance (Fig. 6a–c). The levels of PARP1 and GAPDH were also increased, indicating a higher apoptosis rate in CNE-1 cells upon the transfection of miR-20a-5p-antagomiR (Additional file 3: Figure S2). Taken together, The Rab27B gene does contribute a great deal to the miR-20a-5p’s promoting effect on the NPC radio-resistance.

## Discussion

Radiation therapy is one of the major treatments against tumors as it has the advantages of being non-invasive and well tolerated. However, radiotherapy resistance is also a common occurrence that blocks the effective therapy [27]. The emerging studies have focused on the resistance mechanisms and the involved biological factors [28, 29]. Among the factors involved, miRNAs are reported to be closely associated with tumor radiosensitivity [30–32]. The miR-20a-5p studied here is dysregulated in many human cancers [33–35], and the high level of miR-20a-5p was considered as an indicator of advanced stage, poor prognosis and chemo-therapy resistance. Of note, miR-20a was also found to induce cell radio-resistance by activating the PTEN/PI3 K/Akt signaling pathway in hepatocellular carcinoma [18]. However the knowledge of miR-20a-5p on cancer radio-resistance is still limited, especially in NPC. In this study, we found that miR-20a-5p was involved in NPC radio-resistance, probably by targetingRab27B 3′-UTR. Furthermore we demonstrated that miR-20a-5p-mediated Rab27B repression promoted the invasion and metastasis of NPC cells. Both role and mechanisms of miR-20a-5p and Rab27B in NPC radio-resistance were systematically investigated in cultured cells. Furthermore, the influence of miR-20a-5p and Rab27B on the growth of tumor xenografts was also addressed in nude mice.

Rab27B is a member of Ras-like small GTPases that modulate endocytosis and exocytosis vesicle-trafficking control [36–39]. Rab27B is normally expressed in a large number of secretory cells to regulate secretory pathways [40]. In addition, it is reported that aberrant expression of Rab27B is associated with several types of cancers. For examples, the increased Rab27B expression correlates with lymph node metastasis and is a marker for breast cancer progression [41, 42]. Rab27B can also be recognized as a valuable prognostic indicator for hepatocellular carcinoma patients. In addition, Rab27B regulates invasive tumor growth of colorectal cancer [43], hepatocellular carcinoma [44] and breast cancer [42, 45, 46]. Based on the above studies, Rab27B demonstrates oncogenic function and plays important roles in cancer development. However, the expression of Rab27B, as well as its role in NPC, has barely been investigated. In this study, our data suggest that Rab27B might facilitate the invasive/metastatic phenotypes of NPC, and thus might be treated as a novel marker for clinical diagnosis. We showed that the expression of Rab27B is associated with the radio-resistance of NPC cell lines, which is mediated by miR-20a-5p. Despite that the expression level of γ-H2AX is correlated with the cell apoptosis of NPC cells, other pathways may be activated upon the exposure to the radiation, such as JNK signal pathway [47]. The detailed mechanism for the miR-20a-5p-mediated Rab27B repression of NPC radio-resistance remains to be elucidated.

## Additional files

**Additional file 1: Table S1.** The interested miRNA genes based on the miR-omic analysis. A dozen of miRNAs were differentially expressed in the radio-resistant and radio-sensitive cell lines based on the miR-omic analysis were showed in descending order, has-miR-20a-5p was one of them.

**Additional file 2: Figure S1.** MiR-20a-5p promotes NPC cell viability and sensitizes NPC cells to irradiation. γ-H2AX foci formation was determined in CNE-2 and CNE-1 cells transfected with either miR-20a-5p mimic or miR-20a-5p antagomiR 24 h following irradiation. DAPi staining was performed and micrographs were captured at magnification ×400. The median number of foci formation is presented in bar graphs. Values are presented as the median ± standard deviation.

**Additional file 3: Figure S2.** The protein level of PARP and caspase3 detected by western in NCM, 5PM, NCA, 5PA, si-NC and si-Rab27B transfected CNE-2 and CNE-1 cells respectively.

## Abbreviations

NPC: nasopharyngeal cancer
MiR: MicroRNA
UTR: untranslated region
Rab27B: member RAS oncogene family
5PM: miR-20a-5p
5PA: miR-20a-5p
