# Supplementary figures and images for: MiR–20a-5p promotes radio-resistance by targeting Rab27B in nasopharyngeal cancer cells
Source: Cancer Cell Int. 2017 Mar 1;17:32. doi: 10.1186/s12935-017-0389-7 (PMC5333421; doi:10.1186/s12935-017-0389-7)

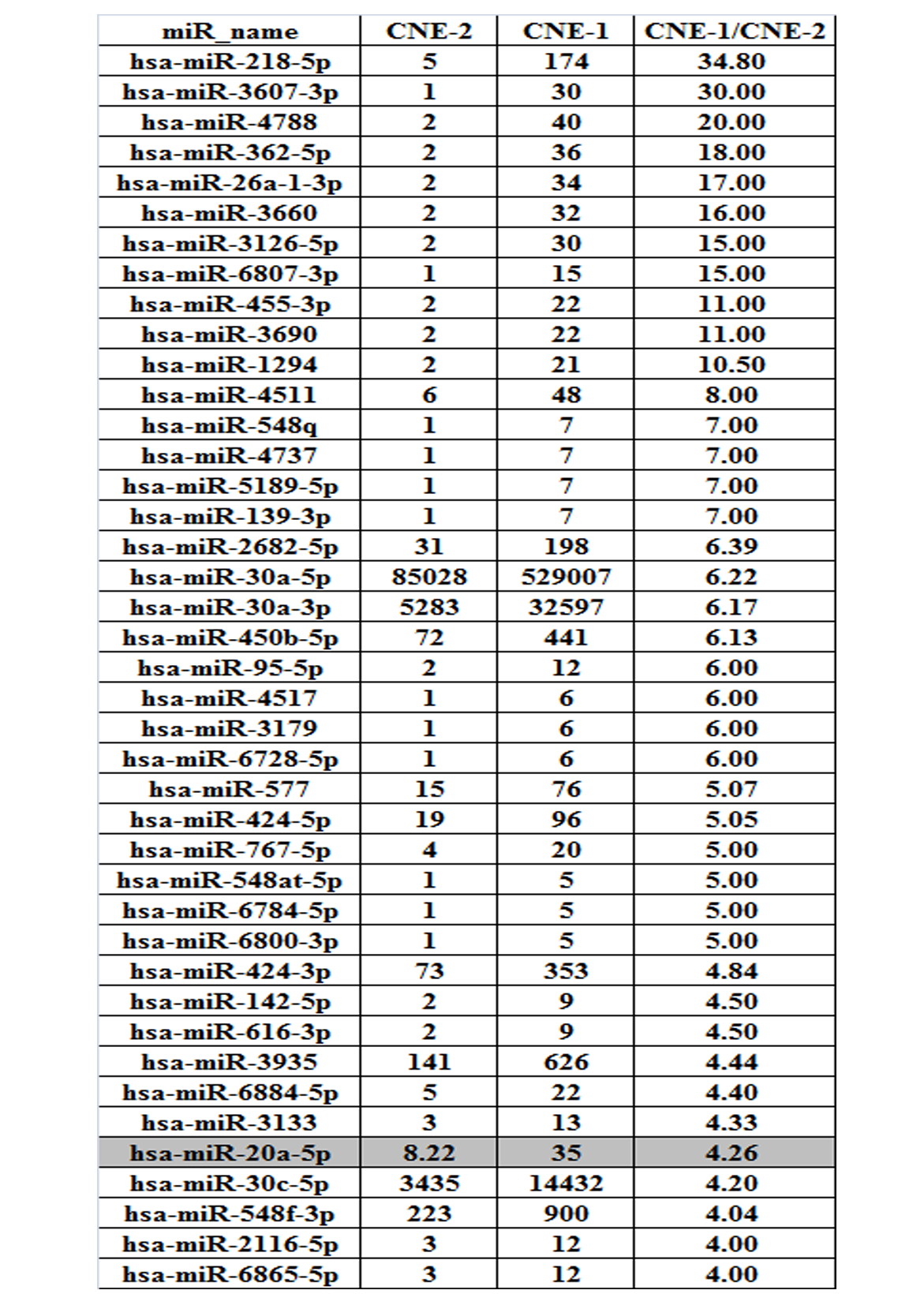

Supplement: Supplementary file 1 — Additional file 1: Table S1. The interested miRNA genes based on the miR-omic analysis. A dozen of miRNAs were differentially expressed in the radio-resistant and radio-sensitive cell lines based on the miR-omic analysis were showed in descending order, has-miR-20a-5p was one of them. [file 12935_2017_389_MOESM1_ESM.tif]

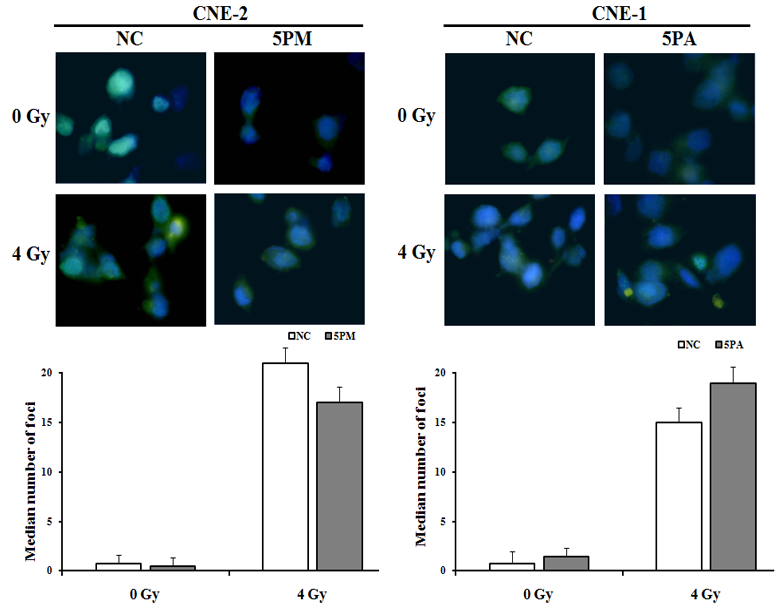

Supplement: Supplementary file 2 — Additional file 2: Figure S1. MiR-20a-5p promotes NPC cell viability and sensitizes NPC cells to irradiation. γ-H2AX foci formation was determined in CNE-2 and CNE-1 cells transfected with either miR-20a-5p mimic or miR-20a-5p antagomiR 24 h following irradiation. DAPi staining was performed and micrographs were captured at magnification ×400. The median number of foci formation is presented in bar graphs. Values are presented as the median ± standard deviation. [file 12935_2017_389_MOESM2_ESM.tif]

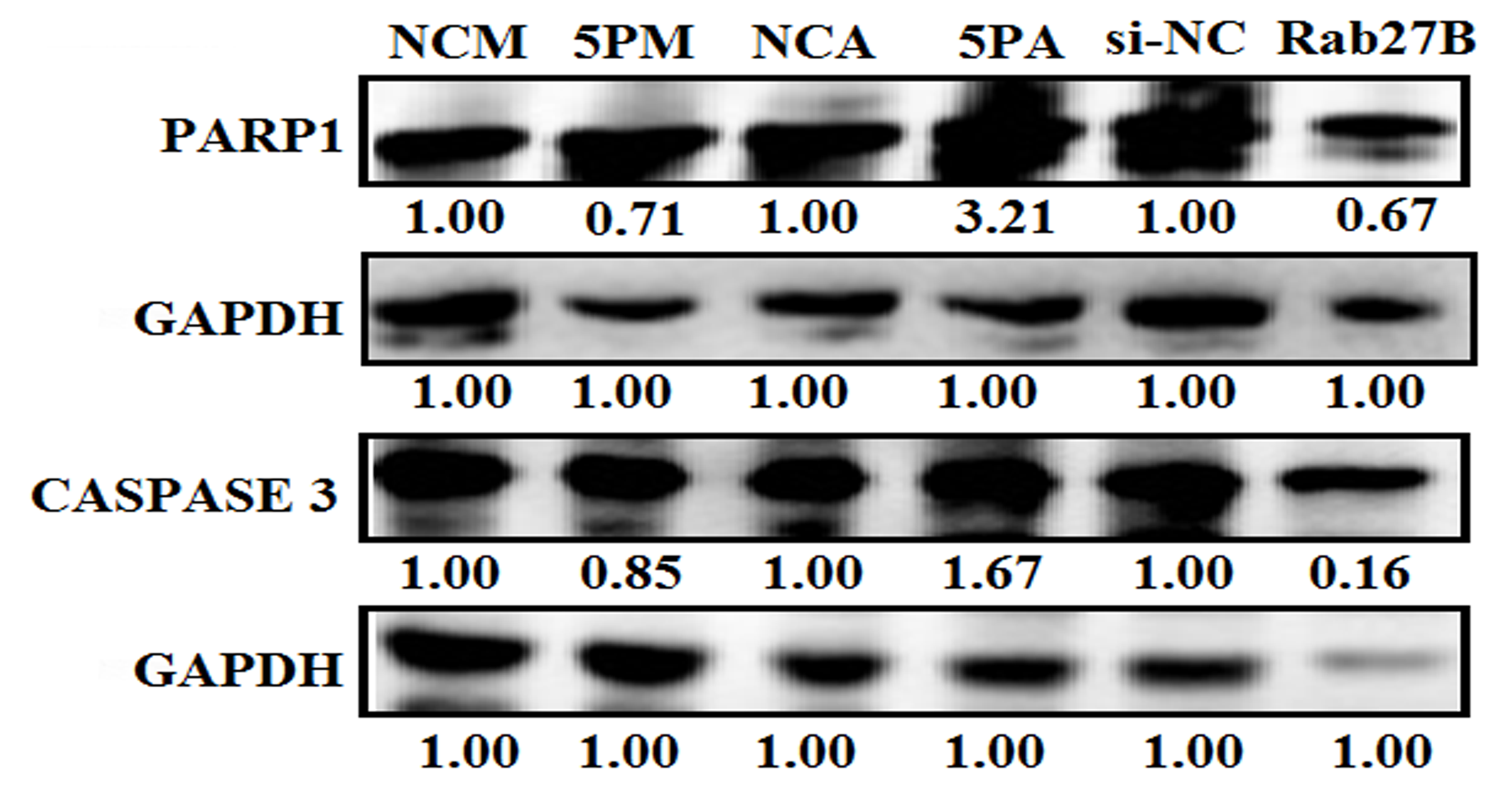

Supplement: Supplementary file 3 — Additional file 3: Figure S2. The protein level of PARP and caspase3 detected by western in NCM, 5PM, NCA, 5PA, si-NC and si-Rab27B transfected CNE-2 and CNE-1 cells respectively. [file 12935_2017_389_MOESM3_ESM.tif]
